# Effects of Resistance Training on Executive Functions of Cognitively Healthy Older Adults: A Systematic Review and Meta-Analysis Protocol

**DOI:** 10.3390/healthcare13020165

**Published:** 2025-01-16

**Authors:** Felipe Jerez-Salas, Christian Campos-Jara, Sergio Araya Sierralta, Daniel Jerez-Mayorga, Rodrigo Ramirez-Campillo, Guido Contreras-Díaz, Vanessa Carrasco-Alarcón, Hugo Martínez-Cortés, Cristián Arellano-Roco, Victoria Hernández-Cifuentes, Falonn Contreras-Osorio

**Affiliations:** 1 Facultad de Salud y Ciencias Sociales, Universidad de Las Américas, Sede Providencia, Manuel Montt 948, Santiago 7500973, Chile; klgo.felipejerez@gmail.com; 2 Exercise and Rehabilitation Sciences Institute, Faculty of Rehabilitation Sciences, Universidad Andres Bello, Santiago 7591538, Chile; christian.campos@unab.cl (C.C.-J.); daniel.jerez@unab.cl (D.J.-M.); 3 Departamento de Educación Física, Universidad de Atacama, Copiapó 1531772, Chile; sergio.araya@uda.cl (S.A.S.); hugo.martinez@uda.cl (H.M.-C.); 4 Strength and Conditioning Laboratory, CTS-642 Research Group, Department Physical Education and Sports, Faculty of Sport Sciences, University of Granada, 18071 Granada, Spain; 5 Exercise and Rehabilitation Sciences Institute, School of Physical Therapy, Faculty of Rehabilitation Sciences, Universidad Andres Bello, Santiago 7591538, Chile; rodrigo.ramirez@unab.cl (R.R.-C.); vihernandez@uc.cl (V.H.-C.); 6 Escuela de Kinesiología, Facultad de Odontología y Ciencias de la Rehabilitación, Universidad San Sebastián, Lago Panguipulli 1390, Puerto Montt 5501842, Chile; guido.contreras@uss.cl; 7 Departamento de Educación Física, Deportes y Recreación, Universidad de La Frontera, Temuco 4811230, Chile; vanessa.carrasco@ufrontera.cl; 8 Departamento de Kinesiología, Universidad Católica del Maule, Talca 3480112, Chile; carellano@ucm.cl

**Keywords:** executive function, exercise, strength, cognition, older adults, meta-analysis

## Abstract

**Background/Objectives**: Aging involves a series of changes in non-pathological age-related conditions, some of which impact the cognitive functioning of older adults. Executive functions are cognitive skills that are often affected in this process, although they have been shown to improve after physical exercise interventions. This protocol aims to describe the procedures that will be carried out in a systematic literature review, including a meta-analysis of the effects of resistance interventions on the main dimensions of executive function in cognitively healthy older adults compared to active or passive control groups. **Methods**: The PRISMA-P guidelines will be followed. Eligibility criteria will be organized based on the PICOS strategy (older adults with normal cognition ≥60 years; chronic resistance interventions ≥4 weeks; active or passive control group; direct measures of executive function). The PubMed, EBSCO, Scopus, and Web of Science databases will be used. The risk of bias and quality of evidence will be measured using RoB2 and GRADE, respectively. The DerSimonian–Laird random effects model will be used for the meta-analysis. The effect size will be calculated using Hedges’ g with a 95% confidence interval and *p* < 0.05 to indicate statistical significance. **Discussion**: The results of the proposed review may be useful to justify the design and implementation of treatment plans based on resistance training for the prevention and management of cognitive changes typical of aging among older adults. PROSPERO registry: CRD42024571127.

## 1. Introduction

As life expectancy increases, the measures addressing the aging process become increasingly important [1]. According to the World Health Organization, by 2025, the proportion of older adults worldwide will reach 22% of the total population [2], along with a life expectancy that will exceed 80 years in some countries [3]. Although aging is a normal process that begins at birth and develops throughout life [4], it involves a series of changes associated with multiple biological functions that determine non-pathological age-related conditions [5], which are considered inevitable [6].

At the level of the brain, it is estimated that the volume of gray and white matter normally decreases between 0.5% and 0.8% per year [7,8], accompanied by tissue changes, decreased metabolic activity, and neurodegeneration [9]. These physiological and morphological changes underlie a lower performance in cognitive domains such as executive functions [10,11], whose impairment is associated with decreased functional performance at older ages [11,12].

Cognition refers to the mental processes that enable the acquisition, processing, storage, and use of knowledge [13] with the aim of thinking, knowing, learning, and being able to carry out activities or tasks with different levels of complexity [14]. Executive functions refer to a set of cognitive processes that regulate goal-directed behavior, playing a key role in monitoring and controlling the mechanisms that mediate the use of information [13]. Cold executive functions (e.g., working memory, inhibition, and cognitive flexibility) include higher-order capacities that allow us to achieve demanding objectives and adapt to novel situations in which we must make decisions, plan, or solve problems considering multiple variables [15,16]. On the other hand, hot executive functions enable the processing of information related to emotion, motivation, and rewards [15]. Therefore, the correct functioning of these skills contributes to healthy aging [16,17], with evolutionary trajectories marked by various factors (e.g., educational level, age, and body mass index) that would allow for predicting the risk of cognitive decline (specifically in these skills) in aging [18,19].

Resistance training has been shown to be an effective alternative intervention for cognitive functioning in older adults [20,21]. Eckardt et al. [22] demonstrated improvements in working memory and inhibitory control in older adults after participating in a 10-week resistance training program. Similarly, Liu et al. [23] showed improvements in executive functioning (selective attention, inhibitory control, working memory, and verbal fluency) in older adults compared to a control group after 12 weeks of resistance training. These improvements have been related to mechanisms underlying the functional and structural changes induced by physical exercise, highlighting an increase in brain blood flow [23], greater activation of the specific regions that control executive functioning [24], and an increase in brain metabolism [25]. Moreover, greater production and release of insulin-like growth factor type 1 (IGF-1) [26] was observed, whose neuroplastic, synaptogenesis, and neuroprotection effects have been related to the neuroplasticity induced by physical exercise [27].

Previous systematic reviews have studied the effects of resistance training interventions on the cognitive functioning of older adults [21,28,29,30,31]. Li et al. [28] conducted a systematic review that examined the effects of resistance training on cognition in older adults with and without mild cognitive impairment. The review by Li et al. [28] suggests that resistance training may benefit some executive function skills (e.g., inhibitory control using the Stroop Color and Word Test), evidenced in six of the nine studies included for these outcome measures in older adults. However, their review considered only a qualitative analysis of studies published in English and Chinese between 2010 and 2017, which was described as a limitation, evidencing possible biases regarding the language and years of publication on which the information search was focused.

On the other hand, Xu et al. [21] conducted a systematic review and meta-analysis whose purpose was to analyze the effects of physical exercise (aerobic or resistance training) on the cognitive functioning of older adults compared to control conditions. This review [20] showed that physical exercise improves cognitive function in older adults for global cognition and specific executive function measures (cognitive flexibility using the Wisconsin Card Sorting Test and Trail Making Test and inhibitory control using the Stroop Color and Word Test). However, it does not provide results for working memory, nor does it present an isolated analysis of strength interventions on executive functions.

In view of previous studies and considering the dimensions in which new information can be added to the existing evidence, the aim of this protocol is to describe the procedures that will be carried out to perform a systematic literature review, which will include a meta-analysis of the effects of interventions that use physical strength exercises on the main dimensions of executive function (working memory, inhibitory control, and cognitive flexibility) in older adults with normal cognition, compared to active or passive control groups. In this research, the literature will be addressed without filtering by language or years of publication to provide an updated, detailed, and unbiased view of the topic under study.

## 2. Materials and Methods

This protocol was developed in accordance with the guidelines established through preferred reporting items for systematic reviews and meta-analysis (PRISMA) protocols (Table S1) [32] and was previously registered in the International Prospective Register of Systematic Reviews (CRD42024571127).

### 2.1. Eligibility Criteria

Table 1 describes the inclusion and exclusion criteria that will be used, taking as a reference the population, intervention, comparator, outcomes, and study design (PICOS) strategy. The minimum age was 60 years since the literature describes a significant increase in neuronal changes related to aging from this age onward [33]. A minimum intervention time of 4 weeks was determined considering the information provided in the previous literature regarding the minimum number of weeks in which improvements in cognitive functioning have been evidenced in older adults after an intervention program based on strength exercises [34]. Studies will be considered that present results on the main dimensions of executive functions (e.g., working memory, inhibition, cognitive flexibility) in older adults with normal cognition, obtained through validated instruments for this population [35]. Due to their importance for synthesis and clinical decision making, both randomized controlled trials (RCTs) and non-randomized controlled trials (non-RCTs) that provide pre- and post-intervention measurements will be included in this study [36]. Studies conducted during the COVID-19 pandemic will not be excluded, as the lockdowns promoted physical activity in controlled, home-based settings [37]. However, these studies must meet all the inclusion criteria outlined in the present protocol.

### 2.2. Search Strategy

Once this protocol is published, the search will be performed in the following databases: Scopus, PubMed, Web of Science, and EBSCO. The references of the selected studies and previous systematic reviews will be analyzed to identify possible studies that meet the established inclusion criteria. The search will be performed by two authors (F.J.S. and C.C.J.) without filtering by publication date, sex, or language. The terms to be used in the search strategy for each database are presented as Supplementary Material (Table S2).

### 2.3. Selection Process

The study selection process will be illustrated by a diagram according to the PRISMA 2020 guidelines [31]. The reasons for exclusion will also be documented for studies that do not meet the PICOS criteria described in this protocol. After initial identification in the databases, studies will be entered into bibliographic management software where duplicate elements will be eliminated automatically and manually if this last step is necessary (F.J.S.). Subsequently, the authors (F.J.S. and F.C.O.) will evaluate the acceptability of the titles and abstracts. In case of an eventual disagreement between the authors who analyze the eligibility of the documents, the opinion of a third author (C.C.J.) will be considered. Finally, the full texts of the articles selected in this first phase will be accessed, and the authors will apply the same procedure to analyze whether they meet all the inclusion criteria. The corresponding authors will request full texts of publications that are not freely available.

### 2.4. Data Extraction and Management

The following information will be identified from each selected study and summarized in tables for qualitative analysis: author(s), year of publication, sample size (total number, experimental group, and control group), sex (male/female), age (years, including the minimum and maximum age range), education (years), cognitive status, health status, and physical activity level. In addition, the main characteristics of the interventions and control groups will be included: intervention characteristics, dropout (number of participants), total duration of interventions (weeks), weekly training frequency, duration of each session (hours), intensity (e.g., percentage of maximum heart rate), characteristics of the control condition, and outcome measures (e.g., Trail Making Test-Part B [32] for cognitive flexibility, N-Back Task [33] for working memory, or Stroop Color and Word Test [34] for inhibition).

The mean and standard deviation before and after the intervention will be recorded for each outcome measure. Data will be extracted independently by two authors (F.J.S. and F.C.O.), who will enter the data into different Microsoft Excel spreadsheets. A third author will verify their agreement and resolve disagreements by re-analyzing the data (C.C.J.). Furthermore, if one or more of the selected papers do not present the data necessary for quantitative analysis, previously established methods in the literature will be used to contact the authors and request the required information [35,36]. The procedure to be used will involve contacting the authors through correspondence once a week for a maximum of 2 weeks by email. In the event of no response or if the data are provided incompletely, the studies will be excluded.

### 2.5. Risk of Bias in Individual Studies

As previously described [37,38,39], the assessment of potential bias will be performed using the second version of the Cochrane risk-of-bias tool. The studies will be classified as minimal risk, of some concern, or high risk after independent analysis by two authors (F.J.S. and F.C.O.) regarding the following domains: randomization process, adherence to planned interventions, handling of missing outcome data, measurement of outcomes, and selection of reported outcomes. Discrepancies will be resolved by a third author (C.C.J.). All studies that meet the inclusion criteria will be analyzed with this tool, the interpretation of which will be part of the discussion of this proposal.

The grading of recommendations, assessment, development, and evaluation (grade) method will be used to categorize the quality of the evidence obtained (high, moderate, low, or very low) for each outcome measure [40].

### 2.6. Meta-Analysis

Once the data are extracted, a meta-analysis will be performed for those outcome measures with at least three studies [41,42,43]. The results to be analyzed will include the three main dimensions of executive functions, i.e., working memory, inhibition, and cognitive flexibility, independently.

The effect size (ES) measure will be Hedges’ g, with a 95% confidence interval. ES will be calculated from the pre- and post-intervention mean and standard deviation values for experimental and control groups using the Der Simonian–Laird methods described in the previous literature [44]. The estimated ES will be classified as follows: <0.2 trivial, 0.2–0.6 small, >0.6–1.2 moderate, >1.2–2.0 large, >2.0–4.0 very large, and >4.0 extremely large [45]. ESs ≥ 0.3 (i.e., an improvement of 3 or more standard deviations from the mean) will be considered outliers because they may affect the validity of the results and, therefore, the interpretations that can be derived from the meta-analysis [46].

For studies involving more than one intervention and only one control group, the sample size of the latter will be divided proportionally to allow for inter-group comparison. Heterogeneity will be measured using the I2 statistic, using the following classification: low (<25%), moderate (25–75%), and high (>75%).

The risk of publication bias will be assessed using Egger’s Test for outcome measures with at least 10 studies [47,48], using the trim and fill method for adjustments [49], and L0 as an estimator for missing studies [50]. A sensitivity analysis will be performed to assess the robustness of the summary estimates (e.g., *p*-value, ES, and I2) [51]. An automated leave-one-out analysis will be performed to assess the impact of each outcome reported by each study within the overall findings. All statistical analyses will be performed using the comprehensive meta-analysis software (version 2, Biostat, Englewood, NJ, USA), considering a statistical significance of *p* ≤ 0.05 [52].

## 3. Discussion

This protocol aims to describe in detail the process by which a systematic literature review with meta-analysis will be carried out to analyze the effects of resistance interventions on the executive functions of older adults with normal cognition compared to control conditions. This will involve an exhaustive search of the scientific literature that describes chronic interventions based on resistance training and its effects on central executive functions (working memory, inhibition, and cognitive flexibility).

Resistance training-based interventions in older adults are presented as a promising alternative given their effects on muscle mass and age-related atrophy processes [53], with their immunological effects against chronic inflammation derived from aging [54], and their cognitive effects [55], being used complementarily with cognitive intervention strategies [56]. Executive functions are a series of skills contained within the broad construct of cognition and closely linked to maintaining functional performance at advanced ages [57,58]. Therefore, understanding the effects of resistance training on these skills could support the implementation of effective interventions for preventing conditions related to typical aging among older adults.

To date, two previous systematic reviews [20,27] addressing the effects of strength exercise interventions in older adults have been found, and several aspects could complement these analyses, which we have included in this proposal. This involves expanding the search to four databases without filters, including outcome measures for the main dimensions of executive function in randomized and non-randomized controlled studies. Analyzing the methodological quality and the limitations of the selected studies will be useful for future research in the field. Likewise, aspects that could contribute to defining future lines of investigation in the area will be highlighted.

The proposed review will be disseminated through scientific publication in a peer-reviewed journal to communicate its results to the widest possible audience, especially to health professionals and people related to the care or support of older adults. These results may be useful for designing and implementing intervention plans aimed at preventing and managing cognitive changes in executive functions, thereby favoring the maintenance of functionality of this population.

## Figures and Tables

**Table 1:** Eligibility criteria.

	Inclusion	Exclusion
Population	Older adults with normal cognition (mean age of the sample ≥60 years), without restrictions regarding physical activity level or sex.	Children, adolescents, or middle-aged adults. Older adults with an underlying medical condition associated with decreased cognitive performance (e.g., neurological pathology, psychiatric disorder, or other).
Intervention	Chronic interventions (lasting ≥4 weeks): Interventions that use resistance training as its sole component, without restriction on the type of exercise (e.g., isokinetic, electromechanical, and isoinertial devices).	Acute interventions (single session). Strength interventions combined or as part of a multi-component program. Interventions that do not involve strength training.
Comparator	Active (e.g., stretching or relaxation) or passive (usual routine) control group.	Absence of a control group.
Outcomes	Direct assessment measures pre- and post-intervention for at least one of the main dimensions of executive function: Working memory, inhibition, or cognitive flexibility, obtained from a validated instrument (e.g., Trail Making Test, Flanker Task, or N-Back Task).	Indirect measures of executive functions (e.g., self-report questionnaires).
Study design	Longitudinal randomized controlled trials (RCTs), and non-RCTs that provide pre- and post-intervention measures.	Case studies, cross-sectional studies, and reviews.

## Data Availability

No new data were created or analyzed in this study. Data sharing is not applicable to this article.

## Supplementary Materials

The following supporting information can be downloaded at: https://www.mdpi.com/article/10.3390/healthcare13020165/s1, Table S1: Checklist PRISMA-P 2015; Table S2: Terms to be used in the search strategy for each database. Ref. [59] was cited in the Supplementary Materials.
