# Supplementary material for: Effects of Resistance Training on Executive Functions of Cognitively Healthy Older Adults: A Systematic Review and Meta-Analysis Protocol
Source: Healthcare (Basel). 2025 Jan 16;13(2):165. doi: 10.3390/healthcare13020165 (PMC11764570; doi:10.3390/healthcare13020165)
Supplement: Supplementary file 1 [file healthcare-13-00165-s001.zip › Table S1.pdf]

Table S1

PRISMA-P (Preferred Reporting Items for Systematic review and Meta-Analysis Protocols) 2015 checklist: recommended items to address in a systematic review protocol*

Section and topic	Item No	Checklist item	Line
**ADMINISTRATIVE INFORMATION**			
Title:			
Identification	1a	Identify the report as a protocol of a systematic review	1-3
Update	1b	If the protocol is for an update of a previous systematic review, identify as such	-
Registration	2	If registered, provide the name of the registry (such as PROSPERO) and registration number	111
Authors:			
Contact	3a	Provide name, institutional affiliation, e-mail address of all protocol authors; provide physical mailing address of corresponding author	7-25
Contributions	3b	Describe contributions of protocol authors and identify the guarantor of the review	233-237
Amendments	4	If the protocol represents an amendment of a previously completed or published protocol, identify as such and list changes; otherwise, state plan for documenting important protocol amendments	-
Support:			
Sources	5a	Indicate sources of financial or other support for the review	238
Sponsor	5b	Provide name for the review funder and/or sponsor	239
Role of sponsor or funder	5c	Describe roles of funder(s), sponsor(s), and/or institution(s), if any, in developing the protocol	239
**INTRODUCTION**			
Rationale	6	Describe the rationale for the review in the context of what is already known	44-106
Objectives	7	Provide an explicit statement of the question(s) the review will address with reference to participants, interventions, comparators, and outcomes (PICO)	99-106
**METHODS**			
Eligibility criteria	8	Specify the study characteristics (such as PICO, study design, setting, time frame) and report characteristics (such as years considered, language, publication status) to be used as criteria for eligibility for the review	107-119
Information sources	9	Describe all intended information sources (such as electronic databases, contact with study authors, trial registers or other grey literature sources) with planned dates of coverage	120-177
Search strategy	10	Present draft of search strategy to be used for at least one electronic database, including planned limits, such that it could be repeated	Supplementary Materials, S2
Study records:			128-140
Data management	11a	Describe the mechanism(s) that will be used to manage records and data throughout the review	

Selection process	11b	State the process that will be used for selecting studies (such as two independent reviewers) through each phase of the review (that is, screening, eligibility and inclusion in meta-analysis)	128-140
Data collection process	11c	Describe planned method of extracting data from reports (such as piloting forms, done independently, in duplicate), any processes for obtaining and confirming data from investigators	141-161
Data items	12	List and define all variables for which data will be sought (such as PICO items, funding sources), any pre-planned data assumptions and simplifications	141-151
Outcomes and prioritization	13	List and define all outcomes for which data will be sought, including prioritization of main and additional outcomes, with rationale	141-161
Risk of bias in individual studies	14	Describe anticipated methods for assessing risk of bias of individual studies, including whether this will be done at the outcome or study level, or both; state how this information will be used in data synthesis	161-173
Data synthesis	15a	Describe criteria under which study data will be quantitatively synthesised	175-199
	15b	If data are appropriate for quantitative synthesis, describe planned summary measures, methods of handling data and methods of combining data from studies, including any planned exploration of consistency (such as I^2, Kendall's τ)	175-199
	15c	Describe any proposed additional analyses (such as sensitivity or subgroup analyses, meta-regression)	192-199
	15d	If quantitative synthesis is not appropriate, describe the type of summary planned	175-179
Meta-bias(es)	16	Specify any planned assessment of meta-bias(es) (such as publication bias across studies, selective reporting within studies)	192-199
Confidence in cumulative evidence	17	Describe how the strength of the body of evidence will be assessed (such as GRADE)	171-173

*** It is strongly recommended that this checklist be read in conjunction with the PRISMA-P Explanation and Elaboration (cite when available) for important clarification on the items. Amendments to a review protocol should be tracked and dated. The copyright for PRISMA-P (including checklist) is held by the PRISMA-P Group and is distributed under a Creative Commons Attribution Licence 4.0.**

From: Shamseer L, Moher D, Clarke M, Ghersi D, Liberati A, Petticrew M, Shekelle P, Stewart L, PRISMA-P Group. Preferred reporting items for systematic review and meta-analysis protocols (PRISMA-P) 2015: elaboration and explanation. BMJ. 2015 Jan 2;349(jan02 1):g7647.
