# Supplementary material for: Effects of Resistance Training on Executive Functions of Cognitively Healthy Older Adults: A Systematic Review and Meta-Analysis Protocol
Source: Healthcare (Basel). 2025 Jan 16;13(2):165. doi: 10.3390/healthcare13020165 (PMC11764570; doi:10.3390/healthcare13020165)
Supplement: Supplementary file 1 [file healthcare-13-00165-s001.zip › Table S2.pdf]

Table S2

Terms to be used in the search strategy according to each database.

Database	Search strategy
EBSCO	AB (“executive function*” OR “cognitive function*” OR cognition OR “inhibitory control” OR “inhibition” OR “interference control” OR “executive control” OR “working memory” OR “updating” OR “executive functioning” OR shifting OR switching OR “cognitive flexibility”) AND AB (“resistance training” OR “strength training” OR “muscle strength” OR “variable resistance training” OR “muscle power” OR “muscle quality” OR strength OR “muscle force” OR “relative strength”) AND AB (aged OR aging OR elderly OR "older person*" OR "older adult*")
PubMed	(((((((((("executive function*"[Title/Abstract]) OR ("cognitive function*"[Title/Abstract])) OR (cognition[Title/Abstract])) OR ("inhibitory control"[Title/Abstract])) OR inhibition[Title/Abstract])) OR “interference control”[Title/Abstract])) OR “executive control”[Title/Abstract])) OR ("working memory"[Title/Abstract])) OR ("updating"[Title/Abstract])) OR (“executive functioning"[Title/Abstract])) OR (shifting[Title/Abstract])) OR (switching[Title/Abstract])) OR ("cognitive flexibility"[Title/Abstract])) AND ((((((((“resistance training”[Title/Abstract]) OR ("strength training"[Title/Abstract])) OR (“muscle strength”[Title/Abstract])) OR (“variable resistance training”[Title/Abstract])) OR ("muscle power"[Title/Abstract])) OR (“muscle quality”[Title/Abstract])) OR (strength[Title/Abstract])) OR ("muscle force"[Title/Abstract]) OR ("relative strength"[Title/Abstract]))) AND (((((((aged[Title/Abstract]) OR (aging[Title/Abstract])) OR (elderly[Title/Abstract])) OR ("older person*"[Title/Abstract])) OR ("older adult*"[Title/Abstract]))
Scopus	(("executive function*") OR ("cognitive function*") OR (cognition) OR ("inhibitory control") OR (inhibition) OR (“interference control”) OR (“executive control”) OR ("working memory") OR ("updating") OR (“executive functioning") OR (shifting) OR (switching) OR ("cognitive flexibility")) AND ((“resistance training”) OR ("strength training") OR (“muscle strength”) OR (“variable resistance training”) OR (“muscle power") OR (“muscle quality”) OR (strength) OR (“muscle force”) OR (“relative strength”)) AND ((aged) OR (aging) OR (elderly) OR ("older person*") OR ("older adult*"))
Web of Science	((TS = (“executive function*” OR “cognitive function*” OR cognition OR “inhibitory control” OR inhibition OR “interference control” OR “executive control” OR “working memory” OR updating OR “executive functioning” OR shifting OR switching OR “cognitive flexibility”)) AND TS = (“resistance training” OR “strength training” OR “muscle strength” OR “variable resistance training” OR “muscle power” OR “muscle quality” OR strength OR “muscle force” OR “relative strength”)) AND TS = (aged OR aging OR elderly OR "older person*" OR " older adult*"))
